# Over-expression of Plk4 induces centrosome amplification, loss of primary cilia and associated tissue hyperplasia in the mouse

**DOI:** 10.1098/rsob.150209

**Published:** 2015-12-23

**Authors:** Paula A. Coelho, Leah Bury, Marta N. Shahbazi, Kifayathullah Liakath-Ali, Peri H. Tate, Sam Wormald, Christopher J. Hindley, Meritxell Huch, Joy Archer, William C. Skarnes, Magdalena Zernicka-Goetz, David M. Glover

**Affiliations:** 1 Department of Genetics, University of Cambridge, Downing Street, Cambridge CB2 3EH, UK; 2 Department of Physiology, Development and Neuroscience, Physiological Laboratory, University of Cambridge, Downing Street, Cambridge CB2 3EG, UK; 3 Centre for Stem Cells and Regenerative Medicine, King's College London, Floor 28, Tower Wing, Guy's Hospital, Great Maze Pond, London SE1 9RT, UK; 4 Wellcome Trust Genome Campus, the Wellcome Trust Sanger Institute, Cambridge, Hinxton CB10 1SA, UK; 5 Henry Wellcome Building of Cancer and Developmental Biology, the Wellcome Trust/Cancer Research UK Gurdon Institute, Tennis Court Road, Cambridge CB2 1QN, UK; 6 Department of Veterinary Medicine, University of Cambridge, Madingley Road, Cambridge CB3 0ES, UK

**Keywords:** primary cilia, Polo-like-kinase-4, skin, tumour development, centrosome amplification, pancreas

## Abstract

To address the long-known relationship between supernumerary centrosomes and cancer, we have generated a transgenic mouse that permits inducible expression of the master regulator of centriole duplication, Polo-like-kinase-4 (Plk4). Over-expression of Plk4 from this transgene advances the onset of tumour formation that occurs in the absence of the tumour suppressor p53. Plk4 over-expression also leads to hyperproliferation of cells in the pancreas and skin that is enhanced in a p53 null background. Pancreatic islets become enlarged following Plk4 over-expression as a result of equal expansion of α- and β-cells, which exhibit centrosome amplification. Mice overexpressing Plk4 develop grey hair due to a loss of differentiated melanocytes and bald patches of skin associated with a thickening of the epidermis. This reflects an increase in proliferating cells expressing keratin 5 in the basal epidermal layer and the expansion of these cells into suprabasal layers. Such cells also express keratin 6, a marker for hyperplasia. This is paralleled by a decreased expression of later differentiation markers, involucrin, filaggrin and loricrin. Proliferating cells showed an increase in centrosome number and a loss of primary cilia, events that were mirrored in primary cultures of keratinocytes established from these animals. We discuss how repeated duplication of centrioles appears to prevent the formation of basal bodies leading to loss of primary cilia, disruption of signalling and thereby aberrant differentiation of cells within the epidermis. The absence of p53 permits cells with increased centrosomes to continue dividing, thus setting up a neoplastic state of error prone mitoses, a prerequisite for cancer development.

## 1. Introduction

The centrosome was first described almost simultaneously by Edouard van Beneden working in Liège and Theodor Boveri in Munich in 1887 (reviewed in [1]) as a structure at the poles of the mitotic spindle that persisted through the life cycle of the cell. Towards the end of the nineteenth century tumour cells were already observed to have multiple spindle poles, and now we know supernumerary centrosomes to be present in a wide range of solid and haematological tumours, including pancreatic, ovarian, colon and prostate cancer, multiple myeloma, non-Hodgkin's and Hodgkin's lymphoma, and acute and chronic myeloid leukaemia [2–7]. Abnormalities have been detected at both early [8–10] and advanced stages of disease where they generally indicate poor prognosis [11]. Amplified centrosomes also correlate with metastasis of head and neck, prostate and breast tumours [12–14]. However, despite these long-known associations, the contribution of centrosomal amplification to oncogenesis remains unclear.

Centrosome number is normally tightly controlled within the cell division cycle. At the core of centrosomes lie the ninefold symmetrical centrioles. Cells enter mitosis with two pairs of orthogonally arranged centrioles that separate to form the two centrosomes at the poles of the spindle. The centrioles disengage upon the completion of mitosis and enable both individual centrioles to initiate the duplication process. Centriole duplication is controlled by Polo-like-kinase-4 (Plk4) that phosphorylates the centriole protein Stil/Ana2 allowing it to recruit Sas6, the core component of the centriole cartwheel [15–18]. Elevating Plk4 expression through its ectopic expression or by eliminating the SCF ubiquitin-protein ligase required for Plk4 destruction results in the formation of multiple centrosomes [19–23].

The loss of centrioles has different consequences in different organisms and tissues. *Drosophila* can tolerate centriole loss in some, but not all, tissues, allowing defective cell divisions to continue [23–27]. However, centrioles also serve as basal bodies, the foundations of cilia and flagellae [28,29], and so are essential to fashion the fly's sensory organs for correct physical coordination [24,30]. In mammalian cells, the physical removal of centrosomes prevents cell cycle progression but eventually centrioles reform by a *de novo* pathway and the cell cycle resumes [31–33]. In the mouse, there is a greater reliance on centrioles to generate primary cilia essential for many types of cell signalling. However, unlike mutants that lack cilia, mutant embryos deficient for the centriole component Sas4 and thereby lacking centrioles exhibit extensive apoptosis associated with elevated p53 expression [34]. Apoptosis was rescued in embryos double mutant for Sas4 and p53, thus identifying a p53-dependent apoptotic pathway triggered by loss of centrioles. This has been further supported by experiments to eliminate Plk4 activity from cultured cells using either an auxin-inducible degradation system or pharmacological inhibition of the enzyme using a small molecule, centrinone [33,35]. In both these cases, loss of Plk4 activity results in loss of centrioles and a p53-dependent arrest of cell cycle progression, the mechanism of which is not understood.

The consequences of Plk4 over-expression also vary in different organisms and in different cell types. Over-expression or stabilization of Plk4 in either cultured *Drosophila* cells or mammalian cells leads to multiple centrosomes [19,21–23,36] and in fertilized *Drosophila* eggs drives the formation of thousands of centrioles at the expense of the normal progression of nuclear division cycles [20]. Strikingly this also happens in unfertilized eggs in which centrioles have been naturally eliminated during oogenesis and in which there is no incoming sperm to provide a basal body. Thus, in this circumstance, centriole formation is entirely driven by Plk4. Moreover, elevated expression of Plk4, and indeed perturbation of centrosome function through several routes, can promote tumourigenesis in flies [37,38].

Correct centrosome behaviour is also required for the development of cerebral cortex of the mammalian brain. Deficiency of any of several centrosome components including Plk4 results in microcephaly [39–41]. To study the effects of elevating Plk4 expression in the mouse brain, Marthiens *et al*. [42] generated transgenic animals in which Plk4 expression could be activated in the central nervous system in response to a tissue-specific Cre-mediated recombination event. This led to a microcephaly-like condition that was ascribed to the poor clustering of amplified centrosomes leading to abnormal mitoses and consequent apoptosis. Cell death could be overcome by removing p53 function, leading to the accumulation of aneuploidy cells that would differentiate rather than proliferate.

We wished to examine the consequences of elevated Plk4 expression, and thereby centrosome amplification, in other tissues in the mouse. To have temporal control on the over-expression of Plk4, we have developed a mouse line in which Plk4 is under the control of a doxycycline-inducible promoter. Induction of Plk4 expression in this mouse leads to an early onset of tumour formation in p53 null mice, behavioural defects suggesting abnormalities of brain development in agreement with a previous study [39], and hyperproliferation of cells in the pancreas and in the skin. Here we focus upon characterizing defects in the skin of these animals and show that elevated Plk4 leads to amplification of centrosomes and loss of primary cilia. Together this leads to both hyperproliferation and uncontrolled differentiation of the basal epidermis. We discuss how these phenotypes can arise and how we might account for the enhancement of these phenotypes when the p53 gene is deleted.

## 2. Results

### 2.1. Elevated Plk4 expression dramatically advances the onset of tumour formation in p53-deficient mice

**Figure 1.**
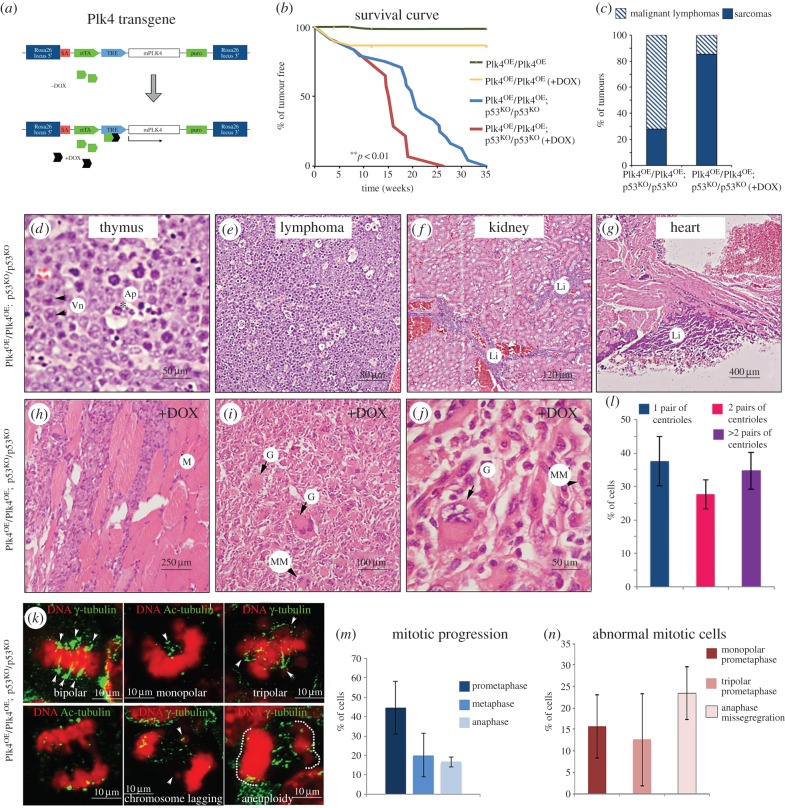
Tumour formation following tetracycline-inducible conditional Plk4 expression. (*a*) Doxycycline associates with the rtTA that binds the TRE, leading to transcriptional activation of Plk4. (*b*) Tumour incidence in Plk4 transgenic mice in wild-type background (Plk4^OE^/Plk4^OE^) or p53 null background (Plk4^OE^/Plk4^OE^; p53^KO^/p53^KO^) with or without treatment with doxycycline (+DOX) to promote Plk4 over-expression. The differences observed between Plk4^OE^/Plk4^OE^; p53^KO^/p53^KO^ (*n* = 24) and Plk4^OE^/Plk4^OE^; p53^KO^/p53^KO^ +DOX (*n* = 14) survival curves are significant (**p < 0.01; Student's *t*-test). (*c*) Proportions of sarcomas and lymphomas. Note that animals that developed sarcomas also showed lymphomas. (*d*) Architecture of thymus and lymph nodes is obliterated and replaced by sheets of large, round cells with vesicular nuclei (Vn, black arrows). Apoptotic cells were also present (Ap, black asterisk). (*e*) Multicentric high-grade large cell lymphoma. (*f*) Kidney has normal architecture but contains multifocal cortical interstitial and sub-capsular infiltrates of large lymphocytes (Li). (*g*) Large sheets of lymphocytes (Li) attached to pericardial surface of heart wall. (*h*–*j*) These tumours are sarcomas isolated from Plk4^OE^/Plk4^OE^; p53^KO^/p53^KO^ mice. Typically, large masses of cells extended into muscle fibre bundles (M) and into attached adipose surrounding nerves and blood vessels. (*i,j*) Higher magnifications of sarcomas showing pleomorphic and anaplastic cells. Examples of giant (G) or multinucleated (MM) cells are indicated. These tumours were found close to the front limbs with high percentage of mitotic cells (an average of 5–10/40× field). (*k*) Paraffin section of samples from sarcomas where stained to reveal γ-tubulin or acetylated-tubulin (green). DNA is shown in red. Three different samples were analysed and showed a high mitotic index (8.55 ± 2.53%, *n* = 1400 cells/sample) in agreement with histological analysis made after H&E staining. (*l*) Proportion of cells that show one pair or two pairs of centrioles per cell or show centriole/centrosome amplification (more than 2 pairs of centrioles). (*m*) Mitotic progression in sarcomas from cryostat sections. (*n*) Proportions of mitotic abnormalities in sarcomas. Quantification in (*l*–*n*) performed in three different sarcomas; 500–1000 cells analysed per sarcoma.

Centrosome amplification has been identified as a marker of poor prognosis of aggressive, drug-resistant tumours in breast, pancreatic and colorectal cancer patients [43,44], but the relationship of centriole amplification to oncogenesis is uncertain. To address this in a model system, we generated a transgenic mouse that allows the inducible over-expression of wild-type mPlk4, the master regulator of centriole duplication. The vector comprised the reverse-tetracycline-controlled transactivator (rtTA) linked to a tetracycline-responsive element (TRE) regulating expression of wild-type mPlk4 and was targeted to the ROSA26 locus. This enabled the inducible and reversible over-expression of Plk4 at any time of development in the resulting transgenic animals or in cultured cells derived from them (figure 1*a*). We designate the homozygous transgenic mouse harbouring the inducible extra copy of wild-type Plk4 as Plk4^OE^/ Plk4^OE^.

As p53 was reported to overcome both the cell cycle arrest associated with loss of centrioles resulting from deletion of Sas4 or degradation or inhibition of Plk4 [33–35] and the cell death resulting from elevated Plk4 expression in the mouse brain [42], we were prompted also to determine the consequences of centrosome amplification induced by Plk4 over-expression in a *p53* knockout (KO) background (from now on p53^KO^). These mice show accelerated tumour formation, behavioural defects and cell hyperproliferation associated with elevated Plk4 expression in several tissues including the pancreas and skin. Here we describe some key features of mice that are expressing elevated levels of Plk4 and focus upon how this affects development of the skin and pancreas.

We first wished to address the effects of Plk4 over-expression upon tumour formation and so carried out parallel studies on the viability of the Plk4^OE^/Plk4^OE^ line with or without the addition of doxycycline (+DOX) to promote Plk4 over-expression. Plk4^OE^/Plk4^OE^ and Plk4^OE^/Plk4^OE^ (+DOX) mice remained healthy during the period of study. Litter sizes were reduced in Plk4^OE^/Plk4^OE^ (+DOX), but tumour formation was not observed during the first 35 weeks (figure 1*b*). We found that approximately one half of Plk4^OE^/Plk4^OE^; p53^KO^/p53^KO^ mice on a doxycycline-free diet developed tumours by the age of 20 weeks and all had tumours by 35 weeks. This time course of tumour appearance parallels previous reports for p53 homozygous null mice [45]. Interestingly, when Plk4 over-expression was induced in the Plk4^OE^/Plk4^OE^; p53^KO^/p53^KO^ mice by the addition of DOX from eight weeks onwards, tumour formation was accelerated; half of the mice developed tumours by 15 weeks and all died within 26 weeks (red line; figure 1*b*).

The tumours arising in Plk4^OE^/Plk4^OE^; p53^KO^/p53^KO^ mice were highly similar whether or not doxycycline was administered and comprised mainly lymphomas and sarcomas (figure 1*c*). The lymphomas invaded a variety of tissues including the thymus, kidney and heart (figure 1*d–g*). However, a wide variety of sarcomas arose more frequently and earlier after Plk4 over-expression. All animals that succumbed to sarcomas also had lymphomas. The sarcomas were typically localized close to the front limbs and developed very rapidly within one week. These tumours had highly pleomorphic and anaplastic cells that displayed a high incidence of mitosis (an average of 5–10 mitotic figures per 40× field) suggesting they are highly malignant (figure 1*h*–*j*) and centrosome amplification was detected in 34.79 ± 5.48% of cells from sarcomas (figure 1*l*). The mitotic figures in these Plk4 over-expressing tumour cells were typically bipolar with multiple centrosomes at the poles (71%). Centrosome clustering was lost in 12.7 ± 10.7% of prometaphase cells that were multipolar (figure 1*k*–*n*). Although we failed to detect any multipolar anaphases, we found chromatid lagging in 23.4 ± 10.6% of anaphase figures (figure 1*n*). It will be of future interest to identify and characterize the mesenchymal cells in which these tumours arise.

We conclude that Plk4 over-expression significantly advances the onset of tumour formation in p53 null mice and that this is associated with an increased frequency of mitoses that generate aneuploid cells characteristic of many tumours.

### 2.2. Elevated Plk4 expression induces hyperproliferation of cells in the pancreas

**Figure 2.**
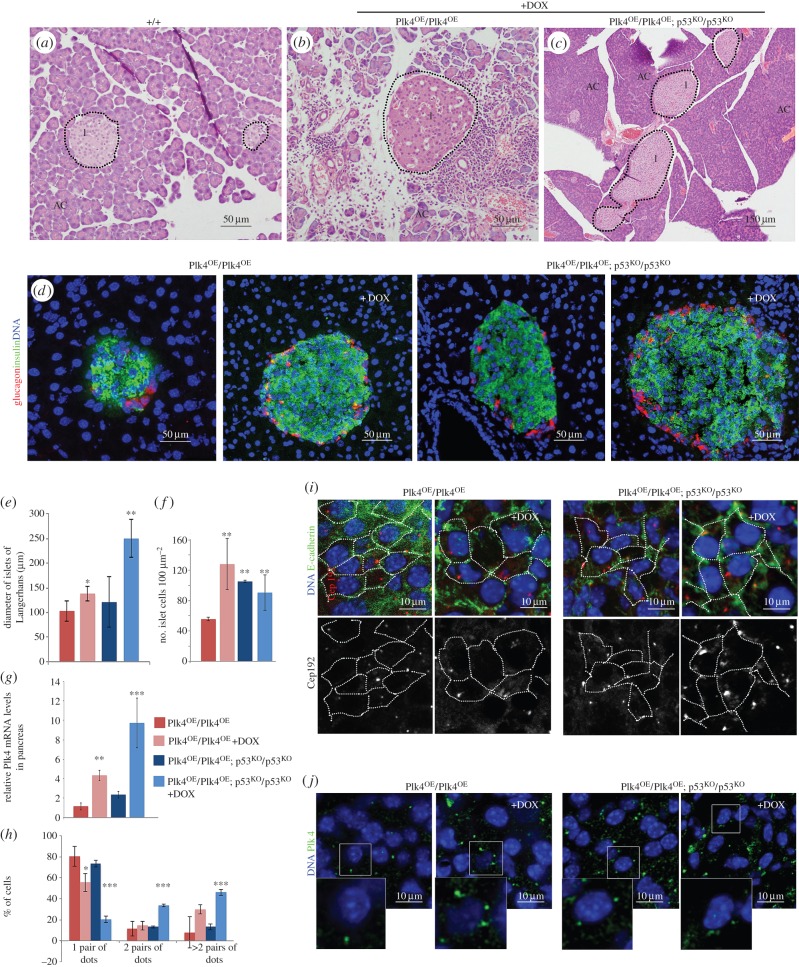
Plk4 over-expression leads to hyperplasia of the pancreatic islets. (*a*–*c*) Haematoxylin–eosin-stained sections of mouse pancreas. (*a*) Control (+/+) pancreas shows normal acinar cells (AC), islets of Langerhans (I) and lobular architecture. (*b*) Pancreas of Plk4^OE^/Plk4^OE^ (+DOX) male has normal lobular architecture, intact acinar cell clusters within lobes and islets of variable size. Lymphocytes are seen surrounding islets and between clusters of acinar cells (black arrows). (*c*) Pancreas from a Plk4^OE^/Plk4^OE^; p53^KO^/p53^KO^ male showing normal lobular architecture and very large islets. (*d*) Immunofluorescence of pancreas cryosections showing β-cells detected by anti-insulin (red) and α-cells detected by anti-glucagon (green) in islets. DNA is blue. (*e*) Diameter of islets (mean ± s.d., *n* = 12) in mice of indicated genotypes without or with (+DOX) doxycycline treatment. (*f*) Density of insulin or glucagon-positive cells (mean ± s.d., *n* = 12) in islets of indicated genotypes without and with doxycline (+DOX) treatment. (*g*) Q-RT-PCR analysis of relative levels of Plk4 transcripts in pancreatic extracts of indicated genotypes without or with (+DOX) doxycycline treatment. Average from three biological samples (three replicates for each). (*h*) Proportion of cells showing 1 (one pair of dots), 2 (two pairs of dots) centrosomes or centrosome amplification (more than two pairs of dots) in pancreatic islets. Centrosomes quantified by counting punctate Cep192 or γ-tubulin staining. (*i*) Pancreas cryosections stained to reveal E-cadherin (green), centrosome component Cep192 (red in merge; white in monochrome) and DNA (blue). Cell borders identified by E-cadherin outlined in monochrome image. Note: increase in the number of centrosomes/cell following treatment with doxycycline (+DOX). (*j*) Pancreas cryosections stained to reveal Plk4 (green) and DNA (blue). Note: increase in the number per cell and size of anti-Plk4-stained dots following treatment with doxycycline (+DOX). Significance was determined by Student's *t*-test. *p < 0.05, **p < 0.01, ***p < 0.005.

The above findings raised the question of whether we could identify any tissues exhibiting common cellular changes that could be associated with events anticipating tumour development particularly in the absence of p53 function. Systematic histological examination of the tissues of Plk4^OE^/Plk4^OE^ mice revealed that although pancreases had normal lobular architecture with intact acinar cell clusters, there was enlargement of the islets of Langerhans when over-expression of Plk4 was induced (figure 2*a*–*c*,*e*). The diameter of the islets was increased by approximately 30% and the density of cells within the islets more than doubled under these conditions (figure 2*e*–*f*). To determine whether this reflected differential proliferation of the major two endocrine cell types, we stained pancreas sections with antibodies against glucagon and insulin to detect α- and β-cells, respectively. This revealed a proportionate increase in the number of both glucagon- and insulin-positive cells following induction of Plk4 over-expression (82.8 ± 6.7% β-cells versus 79.8 ± 6.0% in Plk4^OE^/Plk4^OE^ islets without and with DOX, respectively; figure 2*d*–*f*). Because loss of p53 is known to exacerbate the effects of both decreased and increased centrosome number [33,35,42], we also examined the effects of Plk4 over-expression on the pancreas in p53 null mice. Plk4 over-expression now resulted in a more than doubling in the diameters of the islets although the cell density was not as high as when p53 was present (figure 2*e*,*f*). Again, there was an increase in both α- and β-cells in similar proportions (89.8 ± 3.2% β-cells versus 85.5 ± 3.1% in Plk4^OE^/Plk4^OE^; p53^KO^/p53^KO^ without and with DOX). The size of the islets directly correlated with the levels of Plk4 transcripts (figure 2*g*) and immunostaining of the islets revealed elevated Plk4 protein following treatment of the mice with doxycycline (figure 2*g,h,j*) and an increase in centrosome number (figure 2*h,i*). Plk4 was present in punctate bodies corresponding to centrosomes, and these increased in the number from one such body in non-doxycycline-treated, p53 +/+ cells to four or more in p53 null cells over-expressing Plk4 (figure 2*j*). Thus, elevated expression levels of Plk4 and higher centrosome numbers correlate with hyperproliferation of α- and β-cells in the pancreas that is exacerbated in the absence of p53.

### 2.3. Elevated Plk4 over-expression affects melanocyte differentiation

**Figure 3.**
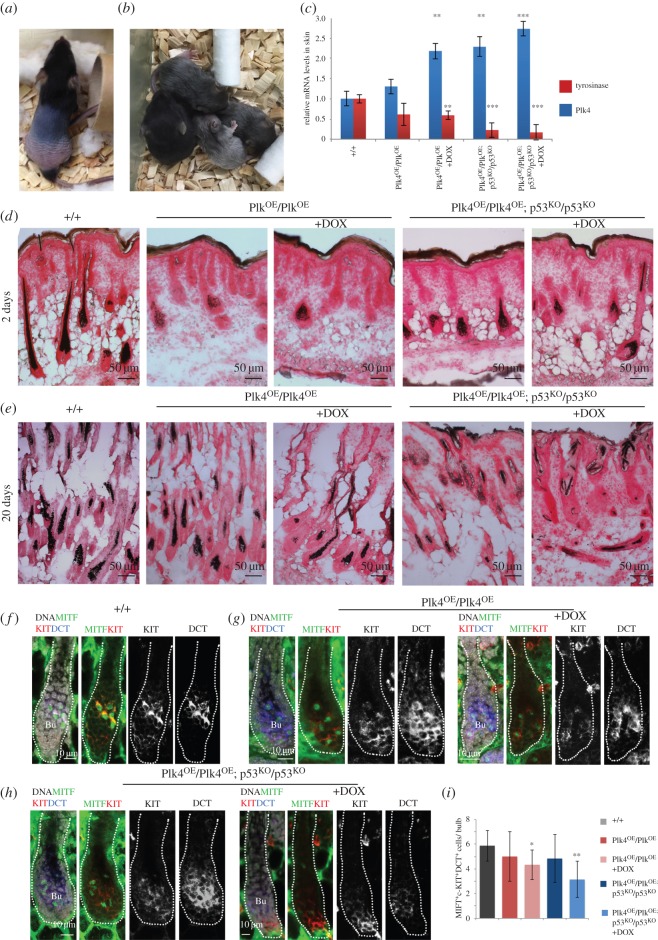
Plk4 over-expression leads to loss of hair and its pigmentation. (*a*) Plk4^OE^/Plk4^OE^; p53^KO^/p53^KO^ mouse exhibiting typical hair loss phenotype. (*b*) Plk4^OE^/Plk4^OE^; p53^KO^/p53^KO^ mice showing varying degrees of greying hair. (*c*) Q-RT-PCR analysis of Plk4 and tyrosinase transcripts in extracts of total back skin from mice of indicated genotypes and treatment indicating mean ± s.e. for three independent biological samples and three replicates/sample (+DOX indicates doxycycline treatment). (*d*) Fontana-Masson staining of cryosections of P2 back skin from mice of indicated genotypes. +DOX indicates treatment with doxycycline. (*e*) Fontana-Masson staining of P20 back skin cryosections from mice of indicated genotypes and treated with doxycycline where indicated (+DOX). (*f*) Anagen hair follicle in wild-type back skin immunostained to reveal melanocyte markers KIT (red in merge; white, monochrome), MITF (green) and DCT (blue in merge; white in monochrome). DNA is represented in white. Differentiated melanocytes identified by triple KIT^+^MITF^+^DCT^+^ staining. (*g*) P2 back skin from Plk4^OE^/Plk4^OE^ mice without or with Plk4 over-expression (+DOX) showing anagen hair follicle immunostained to reveal melanocyte markers KIT (red in merge; white in monochrome), MITF (green) and DCT (blue in merge; white in monochrome). DNA is white in merge. Note reduction in differentiated melanocytes identified by triple KIT^+^MITF^+^DCT^+^-positive immunostaining. (*h*) P2 back skin from Plk4^OE^/Plk4^OE^; p53^KO^/p53^KO^ mice without or with Plk4 over-expression (+DOX) showing hair follicles immunostained for melanocyte markers KIT (red in merge; white in monochrome), MITF (green) and DCT (blue in merge; white in monochrome). DNA is white in merge. Differentiated melanocytes (KIT^+^MITF^+^DCT^+^) are reduced in Plk4^OE^/Plk4^OE^; p53^KO^/p53^KO^ (+DOX) mice. (*i*) Mean numbers ± s.d. of differentiated melanocytes (KIT^+^MITF^+^DCT^+^) per bulb in indicated genotypes and treatments. Significance determined by Student's *t*-test. *p < 0.05, **p < 0.01 and ***p < 0.005.

A striking feature of the Plk4^OE^/Plk4^OE^ mice was skin lesions that include alopecia at the time of weaning followed by regrowth of hair around one month after birth. Animals that did not lose hair had grey coats (figure 3*a,b*). Most of the grey hair did not persist and there was a regain of some black fur within the first month. This phenotype led us to examine the effects of Plk4 over-expression upon melanocytes and the melanin pigment produced by them. Melanocytes are located in the interfollicular epidermis and within hair follicles, and differentiate from neural-crest-derived melanocyte stem cells (MSCs). These MSCs are intermingled with hair follicle stem cells in the bulge and the hair germ. Differentiated melanocytes produce and transfer melanin to the adjacent keratinocytes during the anagen (growth) phase of the cyclical process of hair regeneration (reviewed in [46]). Using Fontana-Masson staining, we found a reduction of melanin granules in the bulge of 2-day-old Plk4^OE^/Plk4^OE^ mice and their apparent absence from follicles, both in a wild-type or p53^KO^/p53^KO^ background (figure 3*d*). We also stained skin sections to examine melanin distribution at 20 days. This revealed a reduction in melanin in Plk4^OE^/Plk4^OE^ mice irrespective of whether animals were treated with doxycycline and that was accentuated if the animals were also null for p53 (figure 3*e*). As an alternative way to assess melanin production, we determined the relative levels of transcripts for tyrosinase, the enzyme catalysing the first step in melanin synthesis and which is expressed in melanocytes [47]. Tyrosinase mRNA was reduced both in untreated Plk4^OE^/Plk4^OE^ mice that show some elevation of basal Plk4 mRNA levels and following doxycycline treatment to induce Plk4 expression. Tyrosinase transcripts were further reduced in a p53 null background (figure 3*c*). Taken together these observations point to reduced melanin biosynthesis in response to elevated Plk4, an effect enhanced in the absence of p53 so accounting for the grey hair of the animals.

We then wanted to determine whether the grey hair was a consequence of reduced melanin production or a reduced number of melanocytes undergoing activation/differentiation. To this end, we carried out immunofluorescence upon cryosections of the back skin of animals 2 days after birth (P2) to detect the MSC population undergoing differentiation (figure 3*f*–*i*). The tyrosine kinase receptor, c-KIT and dopachrome tautomerase (DCT) are hallmarks to identify the MSC population (reviewed in [46]). MSCs co-expressing Kit and microphthalmia-associated transcription factor (MITF), the master regulator of melanocyte differentiation (reviewed in [46]), identify the differentiated melanocyte subpopulation. We found a reduced number of KIT^+^MITF^+^DCT^+^ cells in the bulge when Plk4 was over-expressed, particularly in a p53^KO^/p53^KO^ background (figure 3*i*). This population is responsible for pigmentation of growing hair. However, the number of MSCs (expressing DCT and KIT) in the bulge that will renew the melanocyte population in subsequent hair cycles did not vary significantly between the different genotypes and treatments (an average of 4.35–5 KIT^+^DCT^+^ cells per bulge). This strongly suggests that Plk4 over-expression compromises melanocyte differentiation and subsequent melanin synthesis.

### 2.4. Plk4 over-expression affects cell proliferation in epidermis

The above experiments also revealed a thickening of the epidermis and some apparently invaginated hair follicles when Plk4 levels were elevated in Plk4^OE^/Plk4^OE^; p53^KO^/p53^KO^ mice. Thus, we decided to explore the epidermal phenotype in greater detail. The epidermis is a stratified structure containing self-renewing stem cells within the basal layer that express keratins 5 and 14 (K5, K14) and that through delamination and/or asymmetrical cell division give rise to non-proliferative, spinous and granular layers (expressing keratins 1 and 10 (K1, K10) and Involucrin) and outer layers of terminally differentiated stratum corneum cells (reviewed in [48]). The increased cell density within the epidermal basal layer of Plk4 over-expressing samples (figure 3*e*) prompted us to ask whether cell proliferation was affected as a result of increased levels of Plk4.

**Figure 4.**
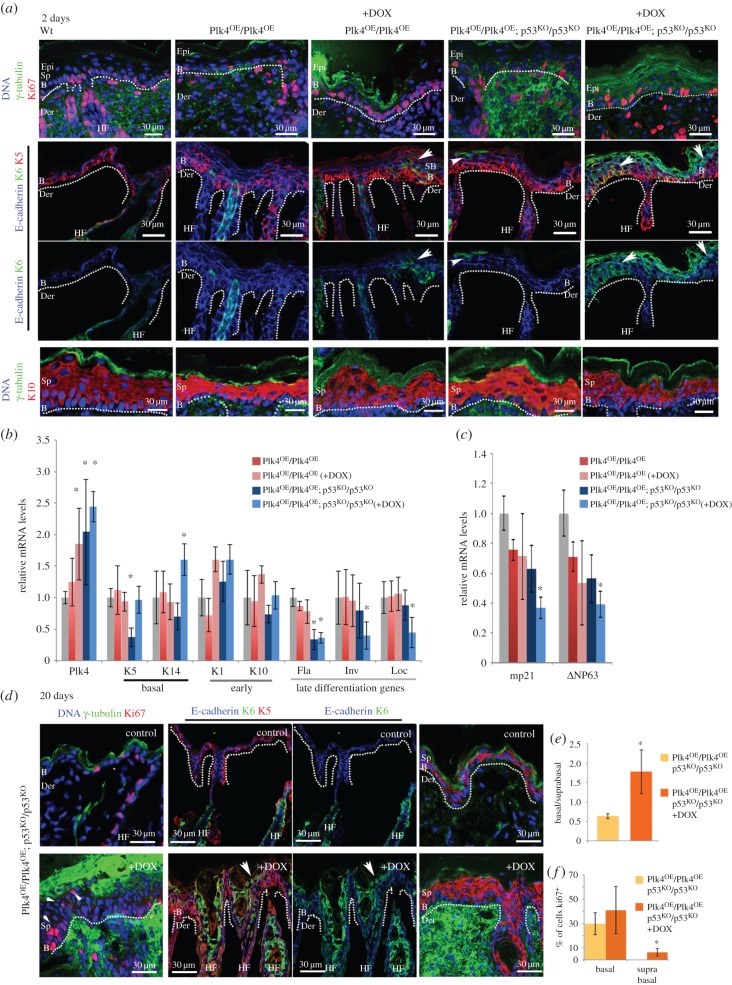
Plk4 over-expression leads to hyperproliferation and abnormal differentiation of cells in suprabasal layers. (*a*) Immunofluorescence of cryosections of P2 back skin from mice of indicated genotypes treated with doxycycline as indicated (+DOX). Note: Ki67 reveals proliferating cells in basal and suprabasal (white asterisk) epidermal layers of Plk4^OE^/Plk4^OE^; p53^KO^/p53^KO^ (+DOX) mice; K6, restricted to hair follicles in controls, is present in epidermis following Plk4 induction (arrowheads). Basal layer marker K5 present throughout epidermis following Plk4 induction; distribution of early differentiation marker, K10 not affected and does not reveal differences between the experimental samples and the control at this stage. B, basal; Der, dermis; Epi, epidermis; Sp, suprabasal; dotted lines, dermo-epidermal border. (*b*) Quantitative RT-PCR assays for levels of indicated transcripts in total back skin from 2 day pups of indicated genotypes and treatment (*n* = 3 and three replicates for each sample). Note: elevated Plk4 transcripts consistently correlate with low expression of late differentiation genes filaggrin (Fla), involucrin (Inv) and loricrin (Loc). (*c*) Quantitative RT-PCR assays as in (*b*) for P21 (mp21) and ΔNP63. (*d*) Immunofluorescence analysis of back skin from 20 day pups of indicated genotypes and treatment. Note: Ki67-positive cells in basal and suprabasal cells (arrowheads) and K6 in multiple layers co-localizing with K5 (white arrow) in Plk4^OE^/Plk4^OE^; p53^KO^/p53^KO^ (+DOX) mice; expanded staining of K5 and K10. (*e*) Ratio of basal to suprabasal cells indicated as mean ± s.d. (*f*) Proportion of cycling cells in epidermis. Mean ± s.d. % of cells showing Ki67-positive immunostaining. Significance determined by Student's *t*-test. *p < 0.05.

To address whether there was an increased number of cycling cells within the basal epidermis, we analysed skin from 2-day-old pups (P2) and 20–day-old pups (P20) using Ki67 as a marker for proliferating cells (figure 4*a*–*c*). In wild-type skin (+/+), Ki67-positive cells are solely found in the basal layer, as these are the only cycling cells in unperturbed circumstances (figure 4*a*). We found Ki67-positive cells in the basal layer did not increase significantly following doxycycline treatment of Plk4^OE^/Plk4^OE^ mice (*n* = 300/ sample; 59.5 ± 19.4% in Plk4^OE^/Plk4^OE^; p53^KO^/p53^KO^ versus 63.9 ± 9.4% in control (+/+)). However, in the skin of Plk4^OE^/Plk4^OE^; p53^KO^/p53^KO^ mice, doxycycline treatment also led to the appearance of Ki67-positive cells in the suprabasal layers (*n* = 425 cells/sample, 7.40 ± 3.90%) within 2 days after birth (asterisk in figure 4*a*). We also examined the distribution of keratin 6 (K6), which is normally restricted to the hair follicles but is also found in the interfollicular epidermis in conditions of hyperplasia the epidermis [49,50]. Strikingly, we found that K6 was widely expressed in basal and suprabasal cells after induction of Plk4 expression in either a wild-type or p53 null background (arrowheads in figure 4*a*). Whereas in normal skin there is a clear separation between basal (K5-positive cells) and suprabasal (K10-positive cells) layers, induction of Plk4 expression in either a wild-type or p53 null background led to an expansion of K5-positive cells. This was particularly evident in Plk4^OE^/Plk4^OE^; p53^KO^/p53^KO^ doxycycline-treated mice at 20 days (figure 4*d*), where both Ki67- and K5-positive cells were significantly expanded to suprabasal layers and K6 staining extended to most of the epidermis and hair follicles. There was also an increase in the number of cells expressing the early differentiation marker, K10.

The above findings were mirrored by the quantitation of mRNA levels for these markers. Over-expression of Plk4 in a p53 null background led to a notable increase in the expression of the basal marker K14 and a reduction in mRNA levels for involucrin, filaggrin and loricrin, markers of late stages of differentiation figure 4*b*). We also found a reduction of the transcript levels of ΔNp63, a p53 family member that controls expansion of epidermal cells and promotes differentiation [51] (figure 4*c*). This, together with a similar reduction in expression of the Cdk inhibitor p21 (mp21; figure 4*c*), is compatible with delayed terminal differentiation [52–54]. In agreement, with these results, percentage of Ki67-positive cells is significantly increased in Plk4^OE^/Plk4^OE^; p53^KO^/p53^KO^ doxycycline-treated mice at 20 days, suggesting increased proliferation sustains the higher number of suprabasal cells in these samples compared to control (figure 4*e,f*).

Taken together our findings indicate that elevated expression of Plk4 results in hyperproliferation of the epidermis. Specifically, we observed an expansion of basal progenitor cells in suprabasal layers and a decreased expression of genes associated with terminal differentiation. These observations accord with the thickening of the epidermis and disrupted hair follicle morphology as a consequence of Plk4 over-expression.

### 2.5. Plk4 over-expression induces centriole over-duplication and primary cilia disappearance in epidermis

**Figure 5.**
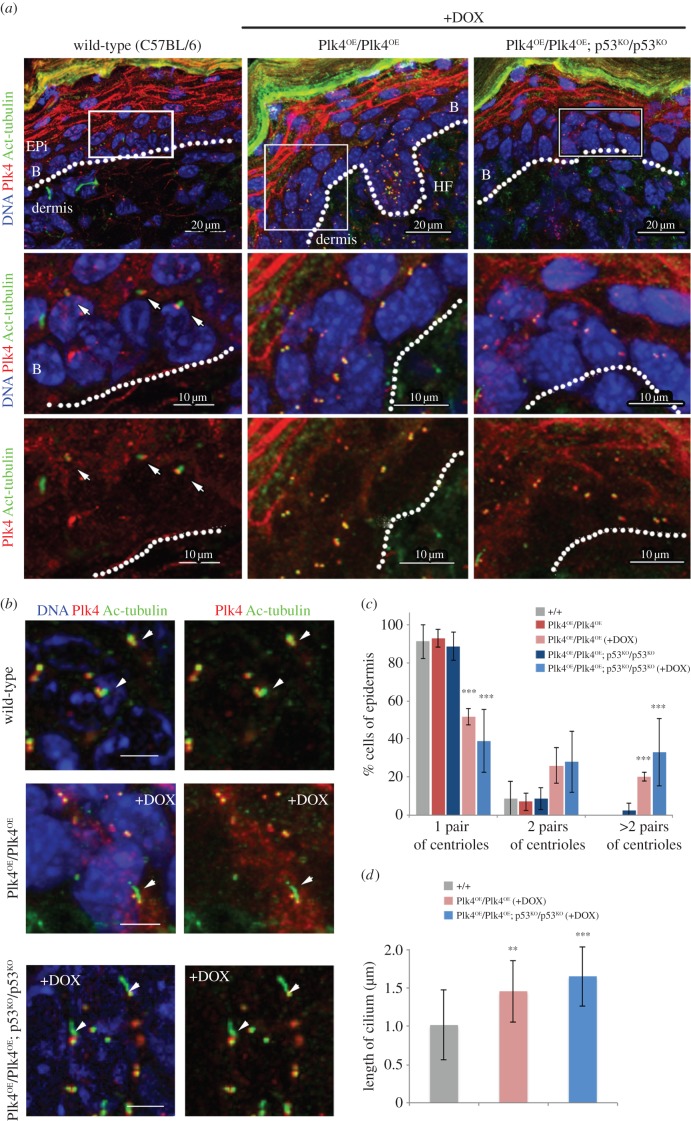
Over-expression of Plk4 leads to multiple centrosomes and loss of primary cilia. (*a*) Immunostaining of back skin of P2 pups of indicated genotypes and treatment to reveal Plk4 (red), acetylated tubulin (green) and DNA (blue). Arrows indicate individual primary cilia (anti-acetylated tubulin) that are apically oriented in basal and suprabasal cells in wild-type control. Note: induction of Plk4 leads to extra centrioles and loss of primary cilia. (*b*) Immunostaining of hair follicles in mice of indicated genotypes and treatment. Note: primary cilia (arrowheads) are still observed in hair follicles after Plk4 induction although they appear longer than in control. (*c*) Quantification of centriole pairs in basal and superbasal layers. Mean ± s.d., *n* = 30–50 cells/condition. (*d*) Quantification of primary cilium length in samples illustrated in (*b*). Mean ± s.d., *n* = 30–50 cells per condition. Significance determined by Student's *t*-test. **p < 0.01, ***p < 0.005.

We next considered whether the alterations we had observed in the regulation of cell proliferation and differentiation in the epidermis could reflect changes mediated by elevated Plk4 upon the centrosomes and/or primary cilia, both of which rely upon centrioles for their correct formation and function. To this end, we stained skin sections to reveal acetylated α-tubulin, a marker of the primary cilia, and Plk4 itself, which associates with centrioles, either in basal bodies or centrosomes. In the skin of wild-type mice, the great majority of cells of the basal epidermis showed bodies of Plk4 staining associated with the base of single primary cilia (figure 5*a*). Over-expression of Plk4 during development led to increased numbers of centrosomes in the basal epidermis and a loss of primary cilia (figure 5*a*,*c*). Loss of primary cilia and mis-positioning of centrosomes was particularly dramatic when Plk4 over-expression occurred in a p53 null background (12.2 ± 4.5% of cells with primary cilia in Plk4^OE^/Plk4^OE^; p53^KO^/p53^KO^ (+DOX) versus 35.3 ± 4.9% in Plk4^OE^/Plk4^OE^; p53^KO^/p53^KO^; figure 5*a*,*c*). We were still able to observe some residual primary cilia in hair follicles even though these cells had extra centrioles (figure 5*b*). However, consistently, only a single primary cilium was formed (figure 5*b*), and these cilia were longer than those in wild-type cells (figure 5*d*). Thus, the effects of elevated Plk4 may differ in different cell types. In the majority of cells of the basal epidermis, Plk4 over-expression results in elevated numbers of centrosomes that form at the expense of primary cilia. This correlates with the increased proliferation of these cells. A primary cilium is still able to form alongside additional centrosomes in some hair follicle cells after Plk4 over-expression but these primary cilia are abnormal in structure.

**Figure 6.**
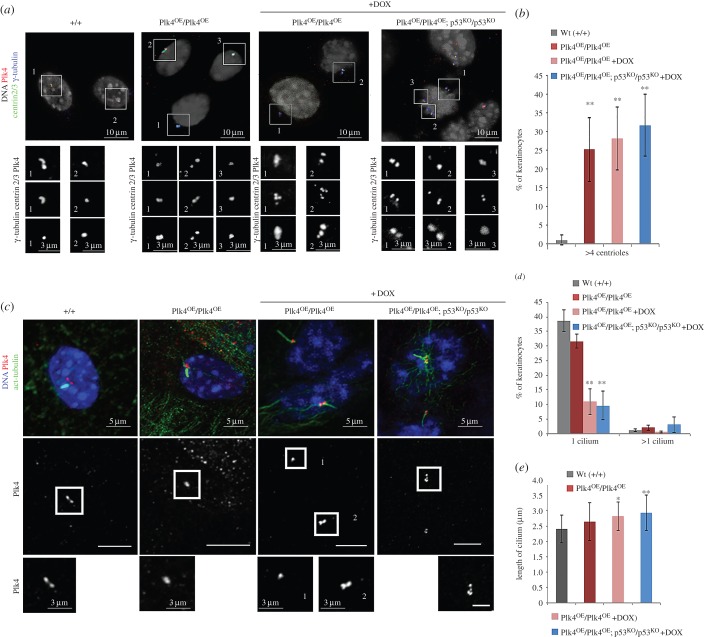
Over-expression of Plk4 leads to multiple centrosomes and loss of primary cilia in primary keratinocytes. (*a*) Primary culture of keratinocytes isolated from Wt, Plk4^OE^/Plk4^OE^ and Plk4^OE^/Plk4^OE^; p53^KO^/p53^KO^ dorsal skin under low calcium conditions. Plk4 over-expression promoted by addition of doxycycline (DOX). Immunostaining reveals Plk4 (red), centrin 2/3 (green) and γ-tubulin (blue). DNA stained with DAPI (white). Individual regions magnified in insets showing example of centriole over-duplication after Plk4 over-expression. (*b*) Quantitation of centrioles in three independent experiments. Mean values ± s.d.; more than 200 cells quantified for each condition. (*c*) *In vitro,* addition of Ca^2+^ induces primary cilia formation in control, Plk4^OE^/Plk4^OE^ and Plk4^OE^/Plk4^OE^; p53^KO^/p53^KO^. In control and Plk4^OE^/Plk4^OE^, 38.70 ± 3.80% and 31.70 ± 2.38% of keratinocytes, respectively, formed a primary cilium after higher Ca^2+^ conditions. If Plk4 is over-expressed, the percentage of keratinocytes showing primary cilia is reduced to 1/3 in Plk4^OE^/Plk4^OE^ compared to controls (11.00 ± 4.38%). Similar results were obtained in Plk4^OE^/Plk4^OE^; p53^KO^/p53^KO^ over-expressing Plk4 (9.66 ± 4.87%). (*d*) Quantitation of percentage of keratinocytes exhibiting one or more primary cilia under indicated conditions. (*e*) Quantitation of primary cilium length under indicated conditions. Significance evaluated by Student's *t*-test and compared to wild-type (*p < 0.05 and **p < 0.01).

We then prepared primary cultures of keratinocytes from our mouse lines as these can be cultured under conditions that permit either cell proliferation or, following the addition of calcium, the formation of cell–cell contacts and cellular differentiation. Induction of Plk4 over-expression following addition of doxycycline to primary Plk4^OE^/Plk4^OE^-derived keratinocytes cultured in low concentrations of calcium reproducibly resulted in centriole over-duplication. (figure 6*a*,*b*). By 48 h after addition of calcium, adherens junctions formed and tight junctions and desmosomes were assembled. Such cells expressed markers of early and late differentiation and grew on top of each other to form a pseudo-three-dimensional epidermis. Under these conditions, a single primary cilium formed at the surface of approximately 40% of cells in either wild-type control cells or in Plk4^OE^/Plk4^OE^ cells without Plk4 induction. Addition of doxycycline to the medium promoted the formation of supernumerary centrioles and the formation of fewer primary cilia (figure 6*b*,*c*). When a primary cilium was formed, there was only one per cell even though multiple centrioles could be present. Moreover, these primary cilia were significantly longer than in wild-type cells or Plk4^OE^/Plk4^OE^ cells that had not been induced to over-express Plk4 (figure 6*d*).

Thus, over-expression of Plk4 results in supernumerary centrosomes both in cells of the basal epidermis and in cultures of primary keratinocytes. In both cases, cells appear to fail to leave the proliferative state and they fail to form primary cilia. This suggests that over-expression of Plk4 leads to an alteration in the balance between proliferation and differentiation of the progenitor epidermal cells. Cilia formation could be compromised either directly by the increased number of centrosomes or as a consequence of a failure of cells to cease cycling sufficiently to enable ciliogenesis. It is known that the primary cilia receive and transmit extracellular signals during development [55–57] and that several ciliary mutants display defects in the commitment of progenitors to differentiate. It is also known that lack of primary cilia in cells of the basal epidermis would compromise the signalling events required to promote their correct differentiation [58,59], so accounting for the defects that we see in cell differentiation in the skin of Plk4 over-expressing mice.

## 3. Discussion

The great majority of tumour cells are both aneuploid and have multiple centrosomes [7,8,60], leading to the suggestion that these properties may be causally related. However, the extent to which chromosome instability resulting from multiple centrosomes contributes to tumour formation is not clear because centrosome clustering at mitosis usually ensures the fidelity of chromosome transmission on bipolar spindles [61]. The transgenic mouse we have generated allows us to induce centriole duplication in response to elevated Plk4 and so begin to address the relationship between multiple centrosomes and tumour formation. Our findings that elevating Plk4 perturbs the balance between proliferation and differentiation in different tissues in a manner exacerbated by loss of the p53 tumour suppressor gives the potential to identify links between supernumerary centrosomes and early steps in tumourigenesis.

Mice deficient for p53 are susceptible to tumour formation [62] and, moreover, the embryonic fibroblasts established from such mice show supernumerary centrosomes [63]. One interpretation of these findings has been that p53 might mediate cell cycle arrest in response to increased centrosome numbers as part of a tetraploidy checkpoint to monitor completion of cytokinesis [64] or by monitoring DNA damage or spindle defects in these cells [65–67]. Others have proposed a direct role for p53 in directly regulating centrosome duplication [68–70]. While it remains difficult to distinguish cause from effect, growing evidence points towards a p53-mediated response to changes in centrosome integrity that receives support from our present study and earlier findings. p53 deletion overcomes either the block to cell progression or apoptosis resulting from depletion of several different centrosome proteins in cultured cells [71] or centriole loss in *Sas4* knockout mouse embryos [34]. Moreover, recent studies have identified a p53 response that is triggered by the inhibition or the destruction of Plk4 that prevents centriole duplication [33,35]. DNA damage, stress, chromosome mis-segregation, prolonged time in mitosis [33,35], nor the Hippo-signalling pathway, recently shown to respond to cytokinesis failure [72], were responsible for triggering this p53-dependent arrest in a senescent-like G1 state in response to centrosome loss. We now show that defects in the pancreas and skin resulting from Plk4 over-expression are enhanced by the loss of p53. We do not know the nature of this p53-dependent response to either gain or loss of centrosomes but it seems possible that it uses a common pathway as both situations can perturb spindle organization and dynamics and cell cycle progression.

The tumour formation that we observe, however, appears dependent upon the loss of p53 function; it is exacerbated by Plk4 over-expression but is not seen following Plk4 over-expression alone. It will be important in future studies to identify the origins of these tumour cells, particularly the sarcomas that appear with increased frequency when Plk4 levels are elevated. That we see tissue hyperplasia rather than neoplasia in the pancreas and skin might reflect the time course of the development of different tumour types in the absence of p53. Usually, p53 null mice first develop lymphomas and most do not survive for long enough for sarcomas to arise. Nevertheless, sarcoma formation predominates in mice in which lymphocytes have been genetically eliminated and it is conceivable that the development of carcinomas, as would occur in the skin, would be masked by these earlier events of lymphoma and then sarcoma formation [73].

The hyperplasia we observe in the pancreas in response to elevated Plk4 and enhanced in the absence of p53 appears to affect the differentiated endocrine cells equally. Thus, the enlarged islets of Langerhans maintain similar proportions of α- and β-cells suggesting that both populations have proliferated and differentiated. In the skin, on the other hand, elevated Plk4 permits proliferating cells to expand into the suprabasal layers of the epidermis where they show inappropriate expression of differentiation markers. This too is enhanced in a p53 background. The shift in the balance of cell proliferation and differentiation could reflect persistence of the centriole as a structure associated with the centrosome and so keeping the cell prepared for cell division. It could also result from a loss of barrier function due to the disruption in the stratified epidermal architecture as occurs, for example, in the inflammatory response (reviewed in [74]). The stratification of the skin occurs when polarized cells of the basal epidermis undergo divisions perpendicular to the basal cell layer to produce the differentiating upper layers [75]. Multiple centrosomes might affect the orientation of mitotic spindles thus perturbing the mechanism that delivers cells from the basal to the suprabasal layers in an orderly manner.

Alternatively, the abnormal differentiation in Plk4 over-expressing, p53 null cells could reflect loss of primary cilia that we observe to reciprocate additional centrosome formation both in cells of the basal epidermis and in cultures of primary keratinocytes established from this skin. The differentiation of cells entering the spinous layer is a Notch signalling-dependent process [76] and a failure of ciliogenesis has been reported to compromise Notch signalling and result in defective epidermal differentiation [58]. Thus, in the absence of sufficient primary cilia, the signalling pathways required for correct differentiation would be defective. Reduced signalling activity was also previously reported in cultured cells expressing elevated levels of Plk4 [77]. However, in the examples studied by these authors, cells with extra centrosomes often formed more than one primary cilium. These cilia were associated with reduced concentrations of signalling molecules and this appeared to be responsible for the reduction in signalling activity. This contrasts to our findings in keratinocytes where supernumerary centrosomes appear to form at the expense of the formation of cilia. The occasional cell that we do find with two primary cilia of increased length seems at odds with these earlier findings. The net outcome of disrupted signalling is the same although in one case it appears to be the consequence of the inefficient operation of the signalling machinery and in the other through the loss of the mechanical apparatus, the cilia themselves.

In addition to the work we now report, another study has described a p53-dependent response to mitotic abnormalities resulting from Plk4 over-expression in the developing mouse brain [42]. These authors described how this led neural stem cells to undertake multipolar mitoses leading to aneuploid cells that were directed to p53-dependent apoptosis. They reported that loss of p53 allowed aneuploid cells to accumulate and differentiate thereby reducing the proportion of proliferating cells and reducing brain size. These consequences seem to differ from the ones we now report in the pancreas and skin where p53 enhances the effects of Plk4 over-expression by allowing cells to proliferate. This suggests that the responses to the supernumerary centrosomes resulting from over-expression of Plk4 might differ in different tissues.

Our study highlights the importance of the p53 pathway in monitoring defects elicited by the elevated expression of Plk4. The increase in centrosome numbers and decrease in primary cilia strongly suggest that the effects we observe reflect the principal function of Plk4 to drive centrosome duplication, although we cannot at this stage exclude an involvement of Plk4 in regulating other aspects of cellular physiology (e.g. [78,79]). Further studies are now required to dissect out the precise manner by which the p53 pathway is triggered together with the mechanisms whereby elevated Plk4 affects cell cycle progression and cellular architecture, how these might affect the differentiation programme of the cell and how this could contribute to tumour development.

## 4. Material and methods

### 4.1. Generation of mESCs inducibly expressing Plk4 from the Rosa26 locus

The rtTA gene and response element (ClonTech) were cloned into a Rosa26 targeting vector [80] upstream of Gateway elements attR1/attR2. mPlk4 cDNA flanked by attL1/attL2 sites was introduced into the vector by Gateway cloning (Life Technology), yielding a Rosa26 targeting vector harbouring tetracycline-inducible cDNA for Plk4. Primers used for subcloning the mousePlk4cDNA were mPlk4RosaFor and mPlk4RosaRev (table 1). The targeting vector was introduced into JM8 mESCs by electroporation, and successful integrations were selected for with 1 µg ml^−1^ puromycin. Correct targeting was confirmed by long-range PCR across the 5′ homology arm of clonal puromycin-resistant cell lines.

**Table 1.** Primers for mPlk4 and genotyping.

mPlk4RosaFor	5′GGGGACAAGTTTGTACAAAAAAGCAGGCTTCATGGCGGCGTGCATCGGGGAGAGGATCGAGGACTTTAAG3′
mPlk4RosaRev	5′GGGGACCACTTTGTACAAGAAAGCTGGGTTTAGGAGTTGGATTAGAAAACATCAGAAGGATGGAAGAAAG3′
Plk4Insert_Forward	5′CCGCGCCTGTCCTTTCTCCC3′
Plk4Insert_Reverse	5′GTCCGGCCAGGACGACGAGG3′
Wt_Rosa_locus	5′GGCAAGCACCACCACTGGCTGGC3′
Wt_Rosa_locus	5′GAAGTGTAACTGTGGACAGAGGAGCC3′
IMR8306_p53Mut_FW	5′CTATCAGGACATAGCGTTGG3′
IMR7778_p53_Rev	5′ATACTCAGAGCCGGCCT'3′
IMR7777_p53_FW	5′ACAGCGTGGTGGTACCTTAT3′

### 4.2. Histological analysis of skin and pancreas

Samples were fixed in 4% paraformaldehyde in PBS at 4°C overnight and then dehydrated and embedded in paraffin wax. Eight micrometre sections were stained with haematoxylin and eosin for histological analysis. Samples from dorsal skin or pancreas were washed in PBS, embedded in OCT compound and kept at −80°C prior to cryosectioning for immunofluorescence.

### 4.3. Fontana-Masson staining

Fontana-Masson staining of backskin cryosections was performed following the protocol provided in the Fontana-Masson kit (Abcam, ab150669).

### 4.4. Culture of kerotinocytes

Mouse keratinocytes were isolated from the backskin of 2-day-old pups from each genetic background using dispase and trypsin. After filtration in 40 µm cell strainers, cells were cultured in low calcium medium (50 µM Ca^2+^) on plates coated with coating matrix (Gibco) as previously described [81]. Once confluency was reached, coverslips were either fixed in −20°C methanol to visualize centrosomes or cultures were shifted to 2 mM Ca^2+^ media for 48 h to analyse cytoskeleton and primary cilium formation.

### 4.5. Antibodies

Primary antibodies were obtained from the following sources: rabbit anti-Cep192 [82] (1 : 1000), rabbit anti-keratin 5 ab52635 (Abcam, 1 : 1000), chicken anti-keratin 5 #905901 (BioLegend, 1 : 1000), rabbit anti-keratin 10 ab76318 (Abcam, 1 : 1000), rabbit anti-keratin 6 #905701 (BioLegend, 1 : 1000), rabbit anti-cKit ab5506 (Abcam), rabbit anti-Ki67 ab15580 (Abcam, 1 : 500), mouse anti-γ-tubulin monoclonal GTU-88 (Sigma, 1 : 200), rabbit anti-γ-tubulin T3559 (Sigma, 1 : 500), rat anti-PLK4 [78] (1 : 1000), mouse anti-centrin2/3 S3332 (Santa Cruz Biotechnology, 1 : 500), guinea-pig anti-insulin ab7842 (Abcam, 1 : 50), mouse monoclonal anti-glucagon ab10988 (Abcam, 1 : 200), mouse monoclonal anti-acetylated-tubulin clone 6-11B-1 (Sigma, 1 : 200), mouse anti-MITF ab80651 (Abcam, 1 : 200), rabbit anti-DCT ab74073 (Abcam, 1 : 200), mouse anti-E-cadherin clone ECDD-2 (Invitrogen, 1 : 200), rat anti-mouse-cKIT clone 2B8 (Biolegend). Secondary antibodies used (1 : 2000 for immunofluorescence) were conjugated with Alexa 488, Alexa 568 or Alexa 647 (Invitrogen) and had minimal cross-reactivity to other species.

### 4.6. Fixation protocol for tissues and cells

For identification of centrosomal proteins by immunofluorescence, preparations were fixed (12 min in ice-cold methanol), quickly rinsed in 1×PBS and then permeabilized in 1×PBS; 0.1% Triton-X100 for three times, for 5 min each. Preparations were blocked with 1×PBS; 0.1% Tween 20; 10% FBS for 1 h, followed by primary antibody incubation in the same solution for at least 2 h at room temperature. Washes were performed in 1×PBS; 0.1% Tween 20 for 30 minutes before the addition of secondary antibody in 1×PBS; 0.1% Tween 20 with 10% FBS. Preparations were washed as previously, mounted in Vectashield with DAPI and sealed.

### 4.7. Microscopy

Images were collected on a Zeiss LSM 510 Meta Laser Scanning Confocal Microscope using 63X/1.4 or 100X/1.4 oil objectives, and the LSM 510 v. 4.2 software. Images were deconvolved using Huygens Professional software; processing and analysis was performed with ImageJ v. 1.50b and Adobe Photoshop CS5.

### 4.8. RT-qPCR conditions

RNA was isolated from the different tissues with RNAqueous Kit (Ambion) and RT-QPCR was performed using Power SYBR^®^ Green RNA-to-CT™ 1-Step Kit (Life Technologies) on a Stepone Plus 96 RT system (Life Technologies) with GAPDH as reference gene. Primers pairs (forward and reverse) used for each gene are indicated in table 2. Specificity was confirmed by subsequent melting curve analysis or gel electrophoresis. Levels of PCR product were expressed as a function of GAPDH. Reactions were performed in triplicate for at least three biological samples and fold changes calculated using the 2^−ΔΔCT^ method.

**Table 2.** Primers for RT-QPCR.

Plk4 forward	5′AGGAGAAACTAATGAGCACCACA3′
Plk4 reverse	5′TGGCTCTCGTGTCAGTCCAA3′
GAPDH forward	5′AAGGTCATCCCAGAGCTGAA3′
GAPDH reverse	5′CTGCTTCACCACCTTCTTGA3′
K1 forward	5′GAACACTAAGCTGGCCCTGGACAT3′
K1 reverse	5′CCTCGGGAGTAACTGGTGGAAACA3′
K5 forward	5′CAGTGTGCCAACCTCCAGAACG3′
K5 reverse	5′AGCCCGCTACCCAAACCAAGAC3′
K10 forward	5′GGAGGGTAAAATCAAGGAGTGGTA3′
K10 reverse	5′TCAATCTGCAGCAGCACGTT3′
K14 forward	5′GACGCCGCCCCTGGTGTG3′
K14 reverse	5′ GGTGGCGATCTCCTGCTC3′
filagrin forward	5′GGAGGCATGGTGGAACTGA3′
filagrin reverse	5′TGTTTATCTTTTCCCTCACTTCTACATC3′
involucrin forward	5′GTCCGGTTCTCCAATTCGTGTTT3′
involucrin reverse	5′GCAATTGGAAGAGAAGCAGCATCAG3′
loricrin forward	5′TCACTCATCTTCCCTGGTGCTT3′
loricrin reverse	5′GTCTTTCCACAACCCACAGGA3′
ΔNp63 forward	5′CTGGAAAACAATGCCCAGAC3′
ΔNp63 reverse	5′GAGGAGCCGTTCTGAATCTG3′
mp21_forward	5′GTGGGTCTGACTCCAGCCC3′
mp21_reverse	5′CCTTCTCGTGAGACGCTTAC3′
tyrosinase forward	5′GCGAAGGCACCGCCCTCTTT3′
tyrosinase reverse	5′TCCCACCAGTGCTGCCCCAA3′

## References

[1] Scheer U 2014 Historical roots of centrosome research: discovery of Boveri's microscope slides in Würzburg. Phil. Trans. R. Soc. B 369, 20130469 (doi:10.1098/rstb.2013.0469) 25047623 10.1098/rstb.2013.0469 PMC4113113

[2] Krämer A, Neben K, Ho AD 2005 Centrosome aberrations in hematological malignancies. Cell Biol. Int. 29, 375–383. (doi:10.1016/j.cellbi.2005.03.004) 15996491 10.1016/j.cellbi.2005.03.004

[3] Lingle WL, Lutz WH, Ingle JN, Maihle NJ, Salisbury JL 1998 Centrosome hypertrophy in human breast tumors: Implications for genomic stability and cell polarity. Proc. Natl Acad. Sci. USA 95, 2950–2955. (doi:10.1073/pnas.95.6.2950) 9501196 10.1073/pnas.95.6.2950 PMC19675

[4] Pihan GA, Purohit A, Wallace J, Knecht H, Woda B, Quesenberry P, Doxsey SJ 1998 Centrosome defects and genetic instability in malignant tumors. Cancer Res. 58, 3974–3985. 9731511

[5] Hsu L-C, Kapali M, DeLoia JA, Gallion HH 2005 Centrosome abnormalities in ovarian cancer. Int. J. Cancer 113, 746–751. (doi:10.1002/ijc.20633) 15499629 10.1002/ijc.20633

[6] Sato N, Mizumoto K, Nakamura M, Nakamura K, Kusumoto M, Niiyama H, Ogawa T, Tanaka M 1999 Centrosome abnormalities in pancreatic ductal carcinoma. Clin. Cancer Res. 5, 963–970. 10353727

[7] Giehl M, Fabarius A, Frank O, Hochhaus A, Hafner M, Hehlmann R, Seifarth W 2005 Centrosome aberrations in chronic myeloid leukemia correlate with stage of disease and chromosomal instability. Leukemia 19, 1192–1197. (doi:10.1038/sj.leu.2403779) 15858613 10.1038/sj.leu.2403779

[8] Lingle WL, Barrett SL, Negron VC, D'Assoro AB, Boeneman K, Liu W, Whitehead CM, Reynolds C, Salisbury JL 2002 Centrosome amplification drives chromosomal instability in breast tumor development. Proc. Natl Acad. Sci. USA 99, 1978–1983. (doi:10.1073/pnas.032479999) 11830638 10.1073/pnas.032479999 PMC122305

[9] Pihan GA, Wallace J, Zhou Y, Doxsey SJ 2003 Centrosome abnormalities and chromosome instability occur together in pre-invasive carcinomas. Cancer Res. 63, 1398–1404. 12649205

[10] Segat D et al. 2010 Pericentriolar material analyses in normal esophageal mucosa, Barrett's metaplasia and adenocarcinoma. Histol. Histopathol. 25, 551–560. 20238294 10.14670/HH-25.551

[11] Yamamoto Y, Matsuyama H, Furuya T, Oga A, Yoshihiro S, Okuda M, Kawauchi S, Sasaki K, Naito K 2004 Centrosome hyperamplification predicts progression and tumor recurrence in bladder cancer. Clin. Cancer Res. 10, 6449–6455. (doi:10.1158/1078-0432.CCR-04-0773) 15475431 10.1158/1078-0432.CCR-04-0773

[12] D'Assoro AB, Lingle WL, Salisbury JL 2002 Centrosome amplification and the development of cancer. Oncogene 21, 6146–6153. (doi:10.1038/sj.onc.1205772) 12214243 10.1038/sj.onc.1205772

[13] Pihan GA, Purohit A, Wallace J, Malhotra R, Liotta L, Doxsey SJ 2001 Centrosome defects can account for cellular and genetic changes that characterize prostate cancer progression. Cancer Res. 61, 2212–2219. 11280789

[14] Reiter R et al. 2009 Centrosome abnormalities in head and neck squamous cell carcinoma (HNSCC). Acta Otolaryngol. 129, 205–213. (doi:10.1080/00016480802165767) 18607971 10.1080/00016480802165767

[15] Ohta M, Ashikawa T, Nozaki Y, Kozuka-Hata H, Goto H, Inagaki M, Oyama M, Kitagawa D 2014 Direct interaction of Plk4 with STIL ensures formation of a single procentriole per parental centriole. Nat. Commun. 5, 5267 (doi:10.1038/ncomms6267) 25342035 10.1038/ncomms6267 PMC4220463

[16] Dzhindzhev NS, Tzolovsky G, Lipinszki Z, Schneider S, Lattao R, Fu J, Debski J, Dadlez M, Glover DM 2014 Plk4 phosphorylates Ana2 to trigger Sas6 recruitment and procentriole formation. Curr. Biol. 24, 2526–2532. (doi:10.1016/j.cub.2014.08.061) 25264260 10.1016/j.cub.2014.08.061 PMC4229625

[17] Kratz A-S, Bärenz F, Richter KT, Hoffmann I 2015 Plk4-dependent phosphorylation of STIL is required for centriole duplication. Biol. Open 4, 370–377. (doi:10.1242/bio.201411023) 25701666 10.1242/bio.201411023 PMC4359743

[18] Moyer TC, Clutario KM, Lambrus BG, Daggubati V, Holland AJ 2015 Binding of STIL to Plk4 activates kinase activity to promote centriole assembly. J. Cell Biol. 209, 863–878. (doi:10.1083/jcb.201502088) 26101219 10.1083/jcb.201502088 PMC4477857

[19] Cunha-Ferreira I, Rodrigues-Martins A, Bento I, Riparbelli M, Zhang W, Laue E, Callaini G, Glover DM, Bettencourt-Dias M 2009 The SCF/Slimb ubiquitin ligase limits centrosome amplification through degradation of SAK/PLK4. Curr. Biol. 19, 43–49. (doi:10.1016/j.cub.2008.11.037) 19084407 10.1016/j.cub.2008.11.037

[20] Rodrigues-Martins A, Riparbelli M, Callaini G, Glover DM, Bettencourt-Dias M 2007 Revisiting the role of the mother centriole in centriole biogenesis. Science 316, 1046–1050. (doi:10.1126/science.1142950) 17463247 10.1126/science.1142950

[21] Rogers GC, Rusan NM, Roberts DM, Peifer M, Rogers SL 2009 The SCF Slimb ubiquitin ligase regulates Plk4/Sak levels to block centriole reduplication. J. Cell Biol. 184, 225–239. (doi:10.1083/jcb.200808049) 19171756 10.1083/jcb.200808049 PMC2654306

[22] Habedanck R, Stierhof Y-D, Wilkinson CJ, Nigg EA 2005 The Polo kinase Plk4 functions in centriole duplication. Nat. Cell Biol. 7, 1140–1146. (doi:10.1038/ncb1320) 16244668 10.1038/ncb1320

[23] Bettencourt-Dias M et al. 2005 SAK/PLK4 is required for centriole duplication and flagella development. Curr. Biol. 15, 2199–2207. (doi:10.1016/j.cub.2005.11.042) 16326102 10.1016/j.cub.2005.11.042

[24] Basto R, Lau J, Vinogradova T, Gardiol A, Woods CG, Khodjakov AL, Raff JW 2006 Flies without centrioles. Cell 125, 1375–1386. (doi:10.1016/j.cell.2006.05.025) 16814722 10.1016/j.cell.2006.05.025

[25] Rodrigues-Martins A, Bettencourt-Dias M, Riparbelli M, Ferreira C, Ferreira I, Callaini G, Glover DM 2007 DSAS-6 organizes a tube-like centriole precursor, and its absence suggests modularity in centriole assembly. Curr. Biol. 17, 1465–1472. (doi:10.1016/j.cub.2007.07.034) 17689959 10.1016/j.cub.2007.07.034

[26] Varmark H, Llamazares S, Rebollo E, Lange B, Reina J, Schwarz H, Gonzalez C 2007 Asterless is a centriolar protein required for centrosome function and embryo development in *Drosophila*. Curr. Biol. 17, 1735–1745. (doi:10.1016/j.cub.2007.09.031) 17935995 10.1016/j.cub.2007.09.031

[27] Conduit PT, Wainman A, Raff JW 2015 Centrosome function and assembly in animal cells. Nat. Rev. Mol. Cell Biol. 16, 611–624. (doi:10.1038/nrm4062) 26373263 10.1038/nrm4062

[28] Fuller MT 1993 The development of Drosophila melanogaster. I. Cold Spring Harbor, NY: Cold Spring Harbor Laboratory Press.

[29] Chevalier RL 1969 The fine structure of campaniform sensilla on the halteres of *Drosophila melanogaster*. J. Morphol. 128, 443–463. (doi:10.1002/jmor.1051280405)

[30] Baker JD, Adhikarakunnathu S, Kernan MJ 2004 Mechanosensory-defective, male-sterile *unc* mutants identify a novel basal body protein required for ciliogenesis in *Drosophila*. Development 131, 3411–3422. (doi:10.1242/dev.01229) 15226257 10.1242/dev.01229

[31] Khodjakov AL, Rieder CL 2001 Centrosomes enhance the fidelity of cytokinesis in vertebrates and are required for cell cycle progression. J. Cell Biol. 153, 237–242. (doi:10.1083/jcb.153.1.237) 11285289 10.1083/jcb.153.1.237 PMC2185537

[32] Mahoney NM, Goshima G, Douglass AD, Vale RD 2006 Making microtubules and mitotic spindles in cells without functional centrosomes. Curr. Biol. 16, 564–569. (doi:10.1016/j.cub.2006.01.053) 16546079 10.1016/j.cub.2006.01.053

[33] Wong YL et al. 2015 Cell biology. Reversible centriole depletion with an inhibitor of Polo-like kinase 4. Science 348, 1155–1160. (doi:10.1126/science.aaa5111) 25931445 10.1126/science.aaa5111 PMC4764081

[34] Bazzi H, Anderson KV 2014 Acentriolar mitosis activates a p53-dependent apoptosis pathway in the mouse embryo. Proc. Natl Acad. Sci. USA 111, E1491–E1500. (doi:10.1073/pnas.1400568111) 24706806 10.1073/pnas.1400568111 PMC3992648

[35] Lambrus BG, Uetake Y, Clutario KM, Daggubati V, Snyder M, Sluder G, Holland AJ 2015 p53 protects against genome instability following centriole duplication failure. J. Cell Biol. 210, 63–77. (doi:10.1083/jcb.201502089) 26150389 10.1083/jcb.201502089 PMC4494000

[36] Holland AJ, Fachinetti D, Zhu Q, Bauer M, Verma IM, Nigg EA, Cleveland DW 2012 The autoregulated instability of Polo-like kinase 4 limits centrosome duplication to once per cell cycle. Genes Dev. 26, 2684–2689. (doi:10.1101/gad.207027.112) 23249732 10.1101/gad.207027.112 PMC3533073

[37] Castellanos E, Dominguez P, Gonzalez C 2008 Centrosome dysfunction in *Drosophila* neural stem cells causes tumors that are not due to genome instability. Curr. Biol. 18, 1209–1214. (doi:10.1016/j.cub.2008.07.029) 18656356 10.1016/j.cub.2008.07.029

[38] Basto R, Brunk K, Vinadogrova T, Peel N, Franz A, Khodjakov AL, Raff JW 2008 Centrosome amplification can initiate tumorigenesis in flies. Cell 133, 1032–1042. (doi:10.1016/j.cell.2008.05.039) 18555779 10.1016/j.cell.2008.05.039 PMC2653712

[39] Martin C-A et al. 2014 Mutations in PLK4, encoding a master regulator of centriole biogenesis, cause microcephaly, growth failure and retinopathy. Nat. Genet. 46, 1283–1292. (doi:10.1038/ng.3122) 25344692 10.1038/ng.3122 PMC4676084

[40] Godinho SA, Pellman D 2014 Causes and consequences of centrosome abnormalities in cancer. Phil. Trans. R. Soc. B 369, 20130467 (doi:10.1098/rstb.2013.0467) 25047621 10.1098/rstb.2013.0467 PMC4113111

[41] Mahmood S, Ahmad W, Hassan MJ 2011 Autosomal recessive primary microcephaly (MCPH): clinical manifestations, genetic heterogeneity and mutation continuum. Orphanet. J. Rare Dis. 6, 39 (doi:10.1186/1750-1172-6-39) 21668957 10.1186/1750-1172-6-39 PMC3123551

[42] Marthiens V, Rujano MA, Pennetier C, Tessier S, Paul-Gilloteaux P, Basto R 2013 Centrosome amplification causes microcephaly. Nat. Cell Biol. 15, 731–740. (doi:10.1038/ncb2746) 23666084 10.1038/ncb2746

[43] Glinsky GV 2006 Genomic models of metastatic cancer: functional analysis of death-from-cancer signature genes reveals aneuploid, anoikis-resistant, metastasis-enabling phenotype with altered cell cycle control and activated Polycomb group (PcG) protein chromatin silencing pathway. Cell Cycle 5, 1208–1216. (doi:10.4161/cc.5.11.2796) 16760651 10.4161/cc.5.11.2796

[44] Finetti P et al. 2008 Sixteen-kinase gene expression identifies luminal breast cancers with poor prognosis. Cancer Res. 68, 767–776. (doi:10.1158/0008-5472.CAN-07-5516) 18245477 10.1158/0008-5472.CAN-07-5516

[45] Harvey M, McArthur MJ, Montgomery CA, Butel JS, Bradley A, Donehower LA 1993 Spontaneous and carcinogen-induced tumorigenesis in p53-deficient mice. Nat. Genet. 5, 225–229. (doi:10.1038/ng1193-225) 8275085 10.1038/ng1193-225

[46] Mort RL, Jackson IJ, Patton EE 2015 The melanocyte lineage in development and disease. Development 142, 620–632. (doi:10.1242/dev.106567) 25670789 10.1242/dev.106567 PMC4325379

[47] Wasmeier C, Hume AN, Bolasco G, Seabra MC 2008 Melanosomes at a glance. J. Cell. Sci. 121, 3995–3999. (doi:10.1242/jcs.040667) 19056669 10.1242/jcs.040667

[48] Hsu Y-C, Li L, Fuchs E 2014 Emerging interactions between skin stem cells and their niches. Nat. Med. 20, 847–856. (doi:10.1038/nm.3643) 25100530 10.1038/nm.3643 PMC4358898

[49] Weiss RA, Eichner R, Sun TT 1984 Monoclonal antibody analysis of keratin expression in epidermal diseases: a 48- and 56-kdalton keratin as molecular markers for hyperproliferative keratinocytes. J. Cell Biol. 98, 1397–1406. (doi:10.1083/jcb.98.4.1397) 6201492 10.1083/jcb.98.4.1397 PMC2113245

[50] Stoler A, Kopan R, Duvic M, Euchs E 1988 Use of monospecific antisera and cRNA probes to localize the major changes in keratin expression during normal and abnormal epidermal differentiation. J. Cell Biol. 107, 427–446. (doi:10.1083/jcb.107.2.427) 2458356 10.1083/jcb.107.2.427 PMC2115222

[51] Danilova N, Sakamoto KM, Lin S 2008 p53 family in development. Mech. Dev. 125, 919–931. (doi:10.1016/j.mod.2008.09.003) 18835440 10.1016/j.mod.2008.09.003

[52] Deng C, Zhang P, Wade Harper J, Elledge SJ, Leder P 1995 Mice Lacking p21CIP1/WAF1 undergo normal development, but are defective in G1 checkpoint control. Cell 82, 675–684. (doi:10.1016/0092-8674(95)90039-X) 7664346 10.1016/0092-8674(95)90039-x

[53] Brugarolas J, Chandrasekaran C, Gordon JI, Beach D, Jacks T, Hannon GJ 1995 Radiation-induced cell cycle arrest compromised by p21 deficiency. Nature 377, 552–557. (doi:10.1038/377552a0) 7566157 10.1038/377552a0

[54] Martín-Caballero J, Flores JM, García-Palencia P, Serrano M 2001 Tumor susceptibility of p21(Waf1/Cip1)-deficient mice. Cancer Res. 61, 6234–6238. 11507077

[55] Bershteyn M, Atwood SX, Woo W-M, Li M, Oro AE 2010 MIM and cortactin antagonism regulates ciliogenesis and hedgehog signaling. Dev. Cell 19, 270–283. (doi:10.1016/j.devcel.2010.07.009) 20708589 10.1016/j.devcel.2010.07.009 PMC3108505

[56] Goetz SC, Anderson KV 2010 The primary cilium: a signalling centre during vertebrate development. Nat. Rev. Genet. 11, 331–344. (doi:10.1038/nrg2774) 20395968 10.1038/nrg2774 PMC3121168

[57] Santos N, Reiter JF 2010 Tilting at nodal windmills: planar cell polarity positions cilia to tell left from right. Dev. Cell 19, 5–6. (doi:10.1016/j.devcel.2010.07.001) 20643343 10.1016/j.devcel.2010.07.001 PMC3998640

[58] Ezratty EJ, Stokes N, Chai S, Shah AS, Williams SE, Fuchs E 2011 A role for the primary cilium in Notch signaling and epidermal differentiation during skin development. Cell 145, 1129–1141. (doi:10.1016/j.cell.2011.05.030) 21703454 10.1016/j.cell.2011.05.030 PMC3135909

[59] Croyle MJ et al. 2011 Role of epidermal primary cilia in the homeostasis of skin and hair follicles. Development 138, 1675–1685. (doi:10.1242/dev.060210) 21429982 10.1242/dev.060210 PMC3074444

[60] Nam H-J, Chae S, Jang S-H, Cho H, Lee J-H 2010 The PI3 K-Akt mediates oncogenic Met-induced centrosome amplification and chromosome instability. Carcinogenesis 31, 1531–1540. (doi:10.1093/carcin/bgq133) 20584748 10.1093/carcin/bgq133

[61] Quintyne NJ, Reing JE, Hoffelder DR, Gollin SM, Saunders WS 2005 Spindle multipolarity is prevented by centrosomal clustering. Science 307, 127–129. (doi:10.1126/science.1104905) 15637283 10.1126/science.1104905

[62] Donehower LA, Harvey M, Slagle BL, McArthur MJ, Montgomery CA, Butel JS, Bradley A 1992 Mice deficient for p53 are developmentally normal but susceptible to spontaneous tumours. Nature 356, 215–221. (doi:10.1038/356215a0) 1552940 10.1038/356215a0

[63] Fukasawa K, Choi T, Kuriyama R, Rulong S, Vande Woude GF 1996 Abnormal centrosome amplification in the absence of p53. Science 271, 1744–1747. (doi:10.1126/science.271.5256.1744) 8596939 10.1126/science.271.5256.1744

[64] Andreassen PR, Lohez OD, Lacroix FB, Margolis RL 2001 Tetraploid state induces p53-dependent arrest of nontransformed mammalian cells in G1. Mol. Biol. Cell 12, 1315–1328. (doi:10.1091/mbc.12.5.1315) 11359924 10.1091/mbc.12.5.1315 PMC34586

[65] Uetake Y, Sluder G 2004 Cell cycle progression after cleavage failure: mammalian somatic cells do not possess a ‘tetraploidy checkpoint’. J. Cell Biol. 165, 609–615. (doi:10.1083/jcb.200403014) 15184397 10.1083/jcb.200403014 PMC2172377

[66] Wong C, Stearns T 2005 Mammalian cells lack checkpoints for tetraploidy, aberrant centrosome number, and cytokinesis failure. BMC Cell Biol. 6, 6 (doi:10.1186/1471-2121-6-6) 15713235 10.1186/1471-2121-6-6 PMC554097

[67] Aylon Y, Michael D, Shmueli A, Yabuta N, Nojima H, Oren M 2006 A positive feedback loop between the p53 and Lats2 tumor suppressors prevents tetraploidization. Genes Dev. 20, 2687–2700. (doi:10.1101/gad.1447006) 17015431 10.1101/gad.1447006 PMC1578695

[68] Tarapore P, Horn HF, Tokuyama Y, Fukasawa K 2001 Direct regulation of the centrosome duplication cycle by the p53-p21Waf1/Cip1 pathway. Oncogene 20, 3173–3184. (doi:10.1038/sj.onc.1204424) 11423967 10.1038/sj.onc.1204424

[69] Tarapore P, Tokuyama Y, Horn HF, Fukasawa K 2001 Difference in the centrosome duplication regulatory activity among p53 ‘hot spot’ mutants: potential role of Ser 315 phosphorylation-dependent centrosome binding of p53. Oncogene 20, 6851–6863. (doi:10.1038/sj.onc.1204848) 11687964 10.1038/sj.onc.1204848

[70] Shinmura K, Bennett RA, Tarapore P, Fukasawa K 2007 Direct evidence for the role of centrosomally localized p53 in the regulation of centrosome duplication. Oncogene 26, 2939–2944. (doi:10.1038/sj.onc.1210085) 17072342 10.1038/sj.onc.1210085

[71] Mikule K, Delaval B, Kaldis P, Jurcyzk A, Hergert P, Doxsey S 2007 Loss of centrosome integrity induces p38-p53-p21-dependent G1-S arrest. Nat. Cell Biol. 9, 160–170. (doi:10.1038/ncb1529) 17330329 10.1038/ncb1529

[72] Ganem NJ, Cornils H, Chiu S-Y, O'Rourke KP, Arnaud J, Yimlamai D, Thery M, Camargo FD, Pellman D 2014 Cytokinesis failure triggers hippo tumor suppressor pathway activation. Cell 158, 833–848. (doi:10.1016/j.cell.2014.06.029) 25126788 10.1016/j.cell.2014.06.029 PMC4136486

[73] Landuzzi L et al. 2014 Genetic prevention of lymphoma in p53 knockout mice allows the early development of p53-related sarcomas. Oncotarget 5, 11 924–11 938. (doi:10.18632/oncotarget.2650) 25426555 10.18632/oncotarget.2650 PMC4322986

[74] Simpson CL, Patel DM, Green KJ 2011 Deconstructing the skin: cytoarchitectural determinants of epidermal morphogenesis. Nat. Rev. Mol. Cell Biol. 12, 565–580. (doi:10.1038/nrm3175) 21860392 10.1038/nrm3175 PMC3280198

[75] Williams SE, Ratliff LA, Postiglione MP, Knoblich JA, Fuchs E 2014 Par3-mInsc and G*α*i3 cooperate to promote oriented epidermal cell divisions through LGN. Nat. Cell Biol. 16, 758–769. (doi:10.1038/ncb3001) 25016959 10.1038/ncb3001 PMC4159251

[76] Estrach S, Cordes R, Hozumi K, Gossler A, Watt FM 2008 Role of the Notch ligand Delta1 in embryonic and adult mouse epidermis. J. Invest. Dermatol. 128, 825–832. (doi:10.1038/sj.jid.5701113) 17960184 10.1038/sj.jid.5701113

[77] Mahjoub MR, Stearns T 2012 Supernumerary centrosomes nucleate extra cilia and compromise primary cilium signaling. Curr. Biol. 22, 1628–1634. (doi:10.1016/j.cub.2012.06.057) 22840514 10.1016/j.cub.2012.06.057 PMC4094149

[78] Coelho PA, Bury L, Sharif B, Riparbelli MG, Fu J, Callaini G, Glover DM, Zernicka-Goetz M 2013 Spindle formation in the mouse embryo requires Plk4 in the absence of centrioles. Dev. Cell 27, 586–597. (doi:10.1016/j.devcel.2013.09.029) 24268700 10.1016/j.devcel.2013.09.029 PMC3898710

[79] Martindill DMJ, Risebro CA, Smart N, Franco-Viseras MDM, Rosario CO, Swallow CJ, Dennis JW, Riley PR 2007 Nucleolar release of Hand1 acts as a molecular switch to determine cell fate. Nat. Cell Biol. 9, 1131–1141. (doi:10.1038/ncb1633) 17891141 10.1038/ncb1633

[80] Vooijs M, Jonkers J, Berns A 2001 A highly efficient ligand-regulated Cre recombinase mouse line shows that LoxP recombination is position dependent. EMBO Rep. 2, 292–297. (doi:10.1093/embo-reports/kve064) 11306549 10.1093/embo-reports/kve064 PMC1083861

[81] Nowak JA, Fuchs E 2009 Isolation and culture of epithelial stem cells. Methods Mol. Biol. 482, 215–232. (doi:10.1007/978-1-59745-060-7_14) 19089359 10.1007/978-1-59745-060-7_14 PMC2760227

[82] Zhu F et al. 2008 The mammalian SPD-2 ortholog Cep192 regulates centrosome biogenesis. Curr. Biol. 18, 136–141. (doi:10.1016/j.cub.2007.12.055) 18207742 10.1016/j.cub.2007.12.055

